# Tracking external introductions of HIV using phylodynamics reveals a major source of infections in rural KwaZulu-Natal, South Africa

**DOI:** 10.1093/ve/vey037

**Published:** 2018-12-11

**Authors:** David A Rasmussen, Eduan Wilkinson, Alain Vandormael, Frank Tanser, Deenan Pillay, Tanja Stadler, Tulio de Oliveira

**Affiliations:** 1 Department of Entomology and Plant Pathology, North Carolina State University, Raleigh, NC, USA; 2 Bioinformatics Research Center, North Carolina State University, Raleigh, NC, USA; 3 KwaZulu-Natal Research Innovation and Sequencing Platform (KRISP), College of Health Sciences, University of KwaZulu-Natal, Durban, South Africa; 4 School of Nursing and Public Health, University of KwaZulu-Natal, Durban, South Africa; 5 Africa Health Research Institute, Durban, South Africa; 6 Research Department of Infection & Population Health, University College London, UK; 7 Division of Infection and Immunity, University College London, UK; 8 Department of Biosystems Science and Engineering, ETH Zürich, Basel, Switzerland; 9 Swiss Institute of Bioinformatics, Lausanne, Switzerland; 10 Centre for the AIDS Programme of Research in South Africa (CAPRISA), Durban, South Africa; 11 Department of Global Health, University of Washington, Seattle, USA

**Keywords:** HIV, molecular epidemiology, phylodynamics, migration

## Abstract

Despite increasing access to antiretrovirals, HIV incidence in rural KwaZulu-Natal remains among the highest ever reported in Africa. While many epidemiological factors have been invoked to explain such high incidence, widespread human mobility and viral movement suggest that transmission between communities may be a major source of new infections. High cross-community transmission rates call into question how effective increasing the coverage of antiretroviral therapy locally will be at preventing new infections, especially if many new cases arise from external introductions. To help address this question, we use a phylodynamic model to reconstruct epidemic dynamics and estimate the relative contribution of local transmission versus external introductions to overall incidence in KwaZulu-Natal from HIV-1 phylogenies. By comparing our results with population-based surveillance data, we show that we can reliably estimate incidence from viral phylogenies once viral movement in and out of the local population is accounted for. Our analysis reveals that early epidemic dynamics were largely driven by external introductions. More recently, we estimate that 35 per cent (95% confidence interval: 20–60%) of new infections arise from external introductions. These results highlight the growing need to consider larger-scale regional transmission dynamics when designing and testing prevention strategies.

## 1. Introduction

While the HIV epidemic hit South Africa relatively late compared with other southern African nations, the epidemic grew explosively in the 1990s from an estimated 0.8 per cent prevalence in 1990 to over 20 per cent in 2000 (UNAIDS 2017). Prevalence nationwide stabilized at around 20 per cent in 2000, but an estimated 7.1 million people are still currently living with HIV in South Africa, more than any other country in the world. Prevalence is highest in the province of KwaZulu-Natal (KZN), where 25–40 per cent of the population is HIV positive in some communities (Zaidi et al. 2013; Kharsany et al. 2015; Shisana et al. 2015). While the increasing lifespan of infected individuals on antiretroviral treatment (ART) can partly explain why prevalence has remained high, incidence also remains alarmingly high at between 3 and 6 per cent per year in KZN (Karim et al. 2011; Nel et al. 2012; Vandormael et al. 2018) and may be even higher in certain high-risk groups such as young women (de Oliveira et al. 2017).

Many factors have been implicated in the explosive growth of the HIV epidemic in southern Africa and KZN in particular, but patterns of human movement in the region have long received special attention (Jochelson et al. 1991; Quinn 1994; Lurie et al. 1997). Migration rates have historically been high in the region, fueled by labor-intensive industries such as mining (Hargrove 2008; Corno and De Walque 2012). Mobility has also increased significantly since the end of Apartheid, and sexual networks are known to be geographically well-connected across long distances (Harrison et al. 2008). But the exact role human movement has played in the dynamics of the epidemic has been debated (Coffee et al. 2007; Deane et al. 2010). Given the rural and rather isolated nature of many KZN communities, it is apparent that some spatial mixing would have been necessary to seed the epidemic in different geographic locations. Beyond seeding local epidemics, increasing mobility and migrant labor may have also played a larger role by placing individuals at an increased risk of infection (Lurie et al. 2003; Welz et al. 2007; Camlin et al. 2010), possibly due to migrants engaging in riskier sexual behavior outside of their home communities (Coffee et al. 2007).

Resolving the contribution of human movement to the HIV epidemic has been challenging due to the difficulty of quantifying the extent to which transmission occurs between individuals within local communities versus new cases being imported through external introductions. While the geographic source of new infections cannot typically be resolved using traditional surveillance data, viral phylogenies can help reveal the source of new infections (Holmes 2004; Pybus and Rambaut 2009; Faria et al. 2011). Broadly speaking, if new infections primarily arise from transmission within local communities, viral samples collected within the community should be more closely phylogenetically related to one another than to samples taken from outside the community; whereas if many new infections are being externally introduced, then local samples will tend to cluster with external samples throughout the tree. Phylogenetics can therefore help reveal the movement of viral lineages within and between different communities. For example, one recent study revealed that a high proportion (∼40%) of new infections in a rural Ugandan community arose from external introductions (Grabowski et al. 2014). Recent phylogenetic studies have also revealed widespread viral movement across larger spatial scales and even across national borders (Gray et al. 2009; Wilkinson et al. 2016).

Here, we explore the role of external introductions in the Africa Health Research Institute’s study population in rural KwaZulu-Natal, where HIV prevalence is ∼30 per cent. Using a phylodynamic approach that couples phylogenetic methods together with epidemiological modeling, we reconstructed epidemic dynamics consistent with estimates from population-based surveillance data. By tracking the movement of viral lineages between populations, we also directly quantified the contribution of local transmission versus external introductions to overall HIV incidence. We found that far from just seeding the local epidemic, external introductions played a large role in sustaining high HIV incidence, thus confirming the important role human mobility and migration have played in the hyper-epidemic setting of KZN.

## 2. Methods

### 2.1 Study population

**Figure 1.**
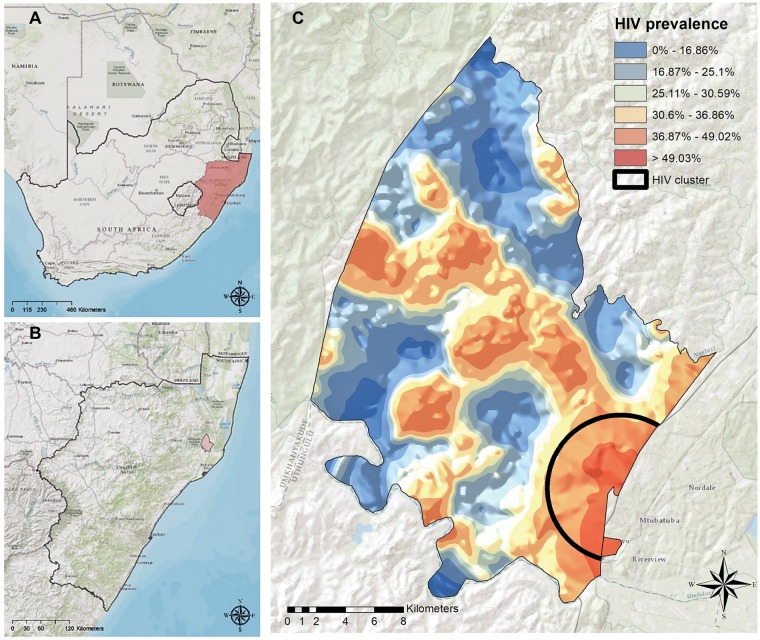
Location of the AHRI study population and prevalence within the area. (A and B) The location of KwaZulu-Natal province and the study area within the province. (C) The spatial variability of HIV prevalence within the study area based on population-based surveillance.

We focused on the Africa Health Research Institute (AHRI) study population in KwaZulu-Natal, South Africa. The AHRI demographic surveillance area (DSA) is located 200 km north of Durban with a mostly rural or peri-urban population (Fig. 1). Demographic data were compiled from the Africa Centre Demographic Information System between 2000 and 2015 (Tanser et al. 2008). There were approximately 87,000 people under surveillance in this population at any given time, with an adult population of males aged 15–54 years and females aged 15–49 years of 55,000. Prevalence and incidence data were also made available from ongoing surveillance.

### 2.2 Population-based HIV surveillance

A population-based HIV survey has been performed within the DSA since 2004, and we compared our phylodynamic estimates of prevalence and incidence against data from this survey. Trained field-workers visit households every 12 months and identify eligible participants aged 15–49 years. After obtaining consent, the field workers then extract blood according to the UNAIDS and WHO Guidelines for Using HIV Testing Technologies in Surveillance. Of the eligible participants contacted, approximately 80 per cent agree to be tested at least once during the study period. Prevalence was determined from the number of individuals who tested HIV-positive during these yearly visits (Vandormael et al. 2018).

We calculated incidence rates using the surveillance data from the subset of eligible participants who had two or more HIV tests, of which the first was a valid HIV-negative result (Vandormael et al. 2014). During the 2004–14 surveillance period, we observed 2,557 seroconversion events for the 17,417 participants in the incidence cohort, irrespective of their residency status or time spent in the DSA. Due to periodic HIV testing, however, we did not know the exact dates of seroconversion events. We therefore used an Monte Carlo-based approach to impute a random seroconversion event between the ith participant’s latest-negative and earliest-positive test dates (Vandormael et al. 2018).

### 2.3 Sequence data and phylogenetic analysis

Partial HIV-1 polymerase (*pol*) sequences were collected as part of population-based surveillance conducted in 2011 and 2014. HIV-1 viral load tests were done on all dried blood spot samples that tested positive by serology (SD Bioline ELISA). Only samples from ART-naive participants with viral loads greater than 10,000 RNA copies/ml were genotyped. More information about the genotyping process is described by Manasa et al. (2016). In total, 1,068 sequence samples from the DSA were included in our analysis. In addition to these samples, we used a large dataset containing 11,289 unique subtype C *pol* sequences described previously by Wilkinson et al. (2016) as a regional background dataset to help identify external introductions. This dataset contained other sequences from South Africa (*n* = 7,739) as well as sequences sampled between 1989 and 2014 from Angola (*n* = 9), Botswana (*n* = 863), the DRC (*n* = 25), Malawi (*n* = 352), Mozambique (*n* = 342), Swaziland (*n* = 47), Tanzania (*n* = 168), Zambia (*n* = 1,476), and Zimbabwe (*n* = 268).

Maximum likelihood (ML) phylogenetic trees were reconstructed using FastTree2 (Price et al., 2010). The ML trees were then dated using Least Squares Dating (To et al. 2015), so that branch lengths were given in units of real calendar time. For dating, we assumed a molecular clock rate of 2.0 × $10^{-3}$, which falls in the center of previously estimated clock rates for subtype C (Wilkinson et al. 2016).

In a preliminary analysis, ML trees were reconstructed from an alignment containing all sequences in the background dataset together with all samples from the AHRI. To identify potential external introductions into the local population, we reconstructed the ancestral location of each internal node in the ML trees using the Fitch parsimony algorithm (Sankoff 1975). External introductions were assumed to occur whenever a child node reconstructed to be in the local population had a parent node reconstructed to be in the external population. The midpoint time between the parent and child node was then used as a proxy for the probable time of introduction.

For the phylodynamic analysis, phylogenetic trees were reconstructed from all AHRI sequences and an equal number of sequences randomly sampled from the background dataset. This was done to reduce the computational cost of fitting the phylodynamic model. To take into account phylogenetic uncertainty and variability across sub-sampled datasets, the phylodynamic analysis was replicated on ten phylogenies each reconstructed from a different set of sequences sub-sampled from the full regional background dataset. Estimates provided in the Results represent an average over these ten phylogenies and sampling replicates (see MCMC details below).

### 2.4 Ethics statement

Ethics permission for the population-based HIV surveillance at the Africa Health Research Institute was obtained from the Biomedical Research Ethics Committee of the College of Health Sciences, University of KwaZulu-Natal (Ethics Nos. BF233/09 and E134/06). All participants in the study provided written informed consent for the analysis of their samples. Sequence samples obtained from HIV positive individuals were obtained from this already-existing study.

### 2.5 Data availability

A representative subset of the HIV pol sequences have been deposited on GenBank with accession numbers MH920641–MH920852. This subset represents 212 of the 1068 total sequences used in this study. The full dataset has been deposited in a secure repository: https://doi.org/10.23664/AC_HIVpol_full1068. Researchers with a clearly demonstrated scientific need may request access to the full dataset by contacting AHRI’s Chief Information Officer, Kobus Herbst at Kobus.Herbst@ahri.org.

### 2.6 Phylodynamic model

Our phylodynamic model divides the host population into a local and an external population. Within the local population, transmission dynamics are modeled using an SIR-type epidemiological model where the number of susceptible (*S*), infected (*I*) or removed (*R*) individuals change over time according to the differential equations:

(1) $$\begin{array}{l} \frac{dS_{l}}{dt}=\mu N_{l}-\beta_{e\to l}(t)\frac{S_{l}}{N_{l}}I_{e}-\beta_{l\to l}(t)\frac{S_{l}}{N_{l}}I_{l}-\mu S_{l}\\ \frac{dI_{l}}{dt}=\beta_{e\to l}(t)\frac{S_{l}}{N_{l}}I_{e}+\beta_{l\to l}(t)\frac{S_{l}}{N_{l}}I_{l}-(\nu +\mu )I_{l}\\ \frac{dR_{l}}{dt}=\nu I_{l}-\mu R_{l} \end{array}$$

The subscripts denote whether a host resides in the local (*l*) or external (*e*) population. Two sources of transmission contribute to new infections within the local population. The first source, the $\beta_{e\to l}(t)\frac{S_{l}}{N_{l}}I_{e}$ term in (1), represents external transmissions into the local population from the external infected population and corresponds to the α(t) terms in Fig. 3. The second source, the $\beta_{l\to l}(t)\frac{S_{l}}{N_{l}}I_{l}$ term in (1), represents transmission within the local population and corresponds to the β(t) terms in Fig. 3. All transmission rates β(t) are allowed to vary over time to accommodate changes in risk behavior, treatment, or other non-modeled factors. The birth rate *μ* and removal rate *ν* are assumed to be constant over time. Upon removal, infected individuals are assumed to no longer be infectious or sexually active.

We assume that the external infected population size *I_e_* grows logistically over time according to an SIS model. The number of susceptible (*S_e_*) and infected (*I_e_*) hosts in the external population change over time as follows:

(2) $$\begin{array}{l} \frac{dS_{e}}{dt}=-\beta_{e\to e}\frac{S_{e}}{N_{e}}I_{e}+\nu I_{e}\\ \frac{dI_{e}}{dt}=\beta_{e\to e}\frac{S_{e}}{N_{e}}I_{e}-\nu I_{e} \end{array}$$

We set the initial infected population size $I_{e}^{init}=1$ in 1975. While this model ignores the considerable spatiotemporal complexity in HIV dynamics within southern Africa, it captures the main trend in HIV dynamics over time—rapidly increasing growth followed by stabilization in prevalence. We therefore view *I_e_* as the effective number of infected individuals living in the region who could have transmitted to an individual living in the local population.

We assume that the time-dependent transmission rates, $\beta_{e\to l}(t)$ and $\beta_{l\to l}(t)$, are constant within a given time interval but can change between intervals in a piecewise-constant manner. To obtain a smoothed estimate of how these parameters change over time, we placed a Gaussian Markov Random Field prior on each parameter. Such GMRF models have been used previously in phylodynamics to prevent large fluctuations in parameter values between neighboring time intervals by penalizing against overly large changes (Minin et al. 2008). The prior probability on a given sequence of time-dependent parameters $\gamma_{1:T}$ is computed as:

(3) $$Pr(\gamma_{1:T}|\tau )\propto \tau^{\frac{T-2}{2}}\;\text{exp}\;\left[-\frac{\tau }{2}\sum_{t=2}^{T-1}\frac{{\left(\gamma_{t+1}-\gamma_{t}\right)}^{2}}{\Delta t}\right],$$

where *τ* is the precision parameter that controls the expected autocorrelation in parameter values between neighboring time intervals. The precision parameter *τ* was inferred separately for each time-dependent parameter. The time interval Δt between change points was fixed at two years between 1990 and 2006. Time-dependent parameters were assumed to be constant after 2006 to prevent overfitting during time periods when the phylogeny was relatively uninformative about changes in epidemic dynamics.

The structured coalescent framework of (Volz 2012) was used to compute the likelihood of the reconstructed phylogenies under our phylodynamic model, which has previously been used to analyze HIV transmission dynamics (Volz et al. 2013; Rasmussen et al. 2014b). The likelihood of a given phylogeny under a general structured coalescent model is:

(4) $$L(T)=\prod_{m=1}^{M-1}\lambda_{ij}(t_{m})\;\text{exp}\;\left[-\int_{s=t_{m}}^{s=t_{m+1}}\sum_{i}^{i\in A(s)}\sum_{j>i}^{j\in A(s)}\lambda_{ij}(s)ds\right].$$

For a tree containing *M* samples, the total likelihood is the product of the likelihood of each of the *M* – 1 coalescent events and the waiting times between events. The likelihood depends on the pairwise coalescent rate $\lambda_{ij}(t)$ at which two lineages *i* and *j* coalesce at time *t*. The total coalescent rate is then computed by summing over all pairs of lineages in the set of lineages A(s) present in the phylogeny at time *s*, which is allowed to change within coalescent intervals due to sampling.

As shown in Volz (2012), under a structured epidemiological model with more than one type of infected individual, the pairwise coalescent rate is:

(5) $$\lambda_{ij}(t)=\sum_{k}^{m}\sum_{l}^{m}\frac{f_{kl}}{I_{k}I_{l}}\left(p_{ik}p_{jl}+p_{il}p_{jk}\right),$$

where *p_ik_* is the probability that lineage *i* is in state *k* and *p_jl_* is the probability that lineage *j* is in state *l*. *I_k_* and *I_l_* are the number of infected individuals in populations *k* and *l*, respectively.

The lineage state probabilities can be computed using a system of differential equations that describe how the probability of lineage *i* being in state *k* evolves backwards in time based on its sampling location:

(6) $$\frac{dp_{ik}}{dt}=\sum_{l}\left(\frac{f_{kl}}{I_{l}}p_{il}-\frac{f_{lk}}{I_{k}}p_{ik}\right)-p_{ik}\sum_{j\neq i}^{j\in A(t)}\sum_{l}^{m}\frac{f_{kl}+f_{lk}}{I_{k}I_{l}}p_{jl}.$$

Here we have modified the equations originally introduced in Volz (2012) by adding the final term in (6), which takes into account how the relative probability of lineage *i* being in state *k* changes conditional on the observation that the lineage has not coalesced with any other lineage *j* in the phylogeny. This was shown by Müller et al. (2017) to improve parameter estimates under asymmetric population dynamics and sampling.

Under our two population HIV model, *f_kl_* represents the transmission rate between populations *k* and *l*. In matrix notation, we have the transmission rate matrix:

(7) $$F(t)\;=\;\left(\begin{array}{ll} \beta_{e\to e}\frac{S_{e}}{N_{e}}I_{e} & \beta_{e\to l}(t)\frac{S_{l}}{N_{l}}I_{e}\\ \beta_{l\to e}I_{l} & \beta_{l\to l}(t)\frac{S_{l}}{N_{l}}I_{l} \end{array}\right).$$

Transmission from the local to the external population allows lineages in the local population to move back into the external population, but the rate $\beta_{l\to e}$ is assumed to be constant over time. Transmission in this direction does not affect the number of infected individuals in the external population, as this is assumed to be much larger than the number of cases exported from the local populations.

We implemented this phylodynamic model in BEAST 2 (Bouckaert et al. 2014) as an add-on package named Marula (Rasmussen 2017). Source code and input XML files that can be used to replicate our analysis are freely available at https://github.com/davidrasm/Marula. We first validated the implementation of our model on mock phylogenies simulated under a stochastic, individual-based version of our base epidemiological model. During these simulations, the full transmission tree was recorded and then subsequently subsampled to produce mock phylogenies. Mock phylogenies were simulated under two parameter regimes where either ∼25 or ∼2.5 per cent of all infections in the local population were due to external introductions, corresponding to the positive and negative controls in Fig. 3D and E, respectively. We verified that the true simulated prevalence, incidence and fraction attributable to external introductions fell within the estimated 95 per cent credible intervals in at least 95 per cent of all years across ten simulations under both parameter regimes.

The posterior distribution of all model parameters and epidemic dynamics were inferred using BEAST’s built-in MCMC sampler. The marginal likelihood of the phylogeny under different models was then computed by taking the harmonic mean of the posterior probabilities sampled by the MCMC algorithm. Due to the large size of the phylogenies, replicate MCMC runs were performed on ten different fixed ML phylogenies reconstructed from different sub-sampled sequence datasets (see above) rather than jointly estimating the full phylogeny while simultaneously fitting the phylodynamic model. Samples from each MCMC replicate were then pooled to obtain posterior estimates averaged over the different ML phylogenies. Each MCMC replicate was run for at least one million iterations.

For inference, weakly informative priors were placed on all estimated parameters (Table 1). In addition, a few demographic parameters were assumed to be known from the AHRI DIS (Table 2). The local population size *N_l_* reflects the adult population size of males 15–54 and females 15–49 years old. The population was assumed to be at demographic equilibrium with a constant birth/death rate of 2.8 per cent per capita each year.

**Table 1.** Prior distributions on all estimated parameters.

Parameter	Name	Prior distribution	Prior values
βl → l	Local transmission rate	Log-normal	μ = −2.3026; σ = 1.0
βe → e	External transmission rate	Log-normal	μ = −0.5065; σ = 1.0
βe → l	External introduction rate	Log-normal	μ = −2.3026; σ = 1.0
βl → e	Local export rate	Log-normal	μ = −2.3026; σ = 1.0
Ne	External pop size	Uniform	min = 55,000 max = 250,000
ν	Removal rate	Log-normal	μ = −2.2769; σ = 0.1
τ	GMRF precision	Gamma	α = 0.01; β = 0.01

**Table 2.** Demographic parameters fixed at constant values.

Parameter	Name	Value
Nl	Local pop size	55,000
μ	Birth/death rate	2.8% per year

### 2.7. Model variants 

In addition to our base phylodynamic model, we consider four other model variants in order to check the robustness of our results to simplifying assumptions made in the base model. In each of these models, the epidemic dynamics in the external population were modeled using (2), but may include different infected classes in the local population.

#### 2.7.1 Hot spot model

The first model variant considers subpopulation structure within the AHRI population due to the clustering of individuals into hot and cold spots of prevalence (Fig. 1C). Spatial scan statistics were used to identify areas with an excess number of HIV cases when compared against a null model assuming a random spatial distribution of cases (Tanser et al. 2009). All other areas were categorized as cold spots. On average, prevalence was >25 per cent in hot spots and <10 per cent in cold spots. The epidemic dynamics in the local population are described by the following system of differential equations:

(8) $$\begin{array}{l} \frac{dS_{h}}{dt}=\mu N_{h}-(\beta_{e\to h}(t)I_{e}+\beta_{h\to h}(t)I_{h}+\beta_{c\to h}(t)I_{c})\frac{S_{h}}{N_{h}}-\mu S_{h}\\ \frac{dS_{c}}{dt}=\mu N_{c}-(\beta_{e\to c}(t)I_{e}+\beta_{c\to c}(t)I_{c}+\beta_{h\to c}(t)I_{h})\frac{S_{c}}{N_{c}}-\mu S_{c}\\ \frac{dI_{h}}{dt}=(\beta_{e\to h}(t)I_{e}+\beta_{h\to h}(t)I_{h}+\beta_{c\to h}(t)I_{c})\frac{S_{h}}{N_{h}}-(\nu +\mu )I_{h}\\ \frac{dI_{c}}{dt}=(\beta_{e\to c}(t)I_{e}+\beta_{c\to c}(t)I_{c}+\beta_{h\to c}(t)I_{h})\frac{S_{c}}{N_{c}}-(\nu +\mu )I_{c}\\ \frac{dR_{h}}{dt}=\nu I_{h}-\mu R_{h}\\ \frac{dR_{c}}{dt}=\nu I_{c}-\mu R_{c} \end{array}$$

Here, the subscripts *h* and *c* indicate whether an individual resides in a hot or cold spot. Based on the geographic location of households within the DSA, approximately 40 per cent of the population lives in hot spots, corresponding to population sizes of about *N*_h_ = 22,000 and *N*_c_ = 33,000.

The same structured coalescent framework as used for the base model can be used to fit this model to the viral phylogenies, but we need to expand the transmission rate matrix *F* used to track the movement of lineages between populations to include hot and cold spots:

(9) $$F(t)\;=\;\left(\begin{array}{lll} \beta_{e\to e}\frac{S_{e}}{N_{e}}I_{e} & \beta_{e\to h}(t)\frac{S_{h}}{N_{h}}I_{e} & \beta_{e\to c}(t)\frac{S_{c}}{N_{c}}I_{e}\\ \beta_{h\to e}I_{h} & \beta_{h\to h}(t)\frac{S_{h}}{N_{h}}I_{h} & \beta_{h\to c}(t)\frac{S_{c}}{N_{c}}I_{h}\\ \beta_{c\to e}I_{c} & \beta_{c\to h}(t)\frac{S_{h}}{N_{h}}I_{c} & \beta_{c\to c}(t)\frac{S_{c}}{N_{c}}I_{c} \end{array}\right).$$

The location of lineages at the tips of phylogenies was assigned based on whether the sampled individual resided in a hot or cold spot.

Parameters and prior distributions for the hotspot model that differ from those used in the base model are given in Table 3. We estimated the time-varying transmission rates $\beta_{e\to c}(t)$ and $\beta_{c\to c}(t)$. The transmission rate from the external population into hot spots was parameterized in terms of a time-invariant scalar $\kappa_{e\to h}$ of the time-varying transmission rate from the external population into cold spots: $\beta_{e\to h}(t)=\kappa_{e\to h}\beta_{e\to c}(t)$. Likewise, the transmission rates within the local population were parameterized in terms of time-invariant scalars of the transmission rate within cold spots, for instance $\beta_{h\to h}(t)=\kappa_{h\to h}\beta_{c\to c}(t)$.

**Table 3.** Parameters and prior distributions for model with hot spots of prevalence.

Parameter	Name	Prior distribution	Prior values
βc → c	Local transmission rate	Log-normal	μ = −2.3026; σ = 1.0
βe → c	External introduction rate	Log-normal	μ = −2.3026; σ = 1.0
κe → h	Transmission scalar	Log-normal	μ = 0.6931; σ = 1.0
κh → h	Transmission scalar	Log-normal	μ = 0.0; σ = 1.0
κh → c	Transmission scalar	Log-normal	μ = 0.0; σ = 1.0
κc → h	Transmission scalar	Log-normal	μ = 0.0; σ = 1.0
Nh	Hot spot pop size	Fixed	22,000
Nc	Cold spot pop size	Fixed	33,000

#### 2.7.2 Time-varying removal model

The second model is very similar to the base model but allows for individuals to remain in the infected class longer as the epidemic progresses due to decreasing removal rates, which mimics improvements in clinical care or treatment over time. We allowed the duration of infection to gradually lengthen by shifting our prior on the removal rate *ν* to lower values over time (Table 4).

**Table 4.** Prior distributions on time-varying removal rates and the implied mean duration of infection.

Parameter	Time period	Prior distribution	Prior values	Mean duration (years)
ν	<2002	Log-normal	μ = −2.2769; σ = 0.1	9.75
ν	2002–4	Log-normal	μ = −2.6208; σ = 0.1	13.75
ν	2004–6	Log-normal	μ = −2.8762; σ = 0.1	17.75
ν	2006–14	Log-normal	μ = −3.0795; σ = 0.1	21.75

#### 2.7.3 ART model

Our third model variant allows infected individuals in the local population to initiate ART after 2004, when widespread ART coverage began in South Africa. Under this model, the epidemic dynamics in the local population are described by the following system of differential equations:

(10) $$\begin{array}{l} \frac{dS_{l}}{dt}=\mu N_{l}-(\beta_{e\to l}(t)I_{e}+\beta_{l\to l}(t)I_{l}+\beta_{t\to l}(t)I_{t})\frac{S_{l}}{N_{l}}-\mu S_{l}\\ \frac{dI_{l}}{dt}=(\beta_{e\to l}(t)I_{e}+\beta_{l\to l}(t)I_{l}+\beta_{t\to l}(t)I_{t})\frac{S_{l}}{N_{l}}-(\nu +\mu +\gamma_{t})I_{l}\\ \frac{dI_{t}}{dt}=\gamma_{t}I_{l}-(\nu_{t}+\mu )I_{t}\\ \frac{dR_{l}}{dt}=\nu I_{l}+\nu_{t}I_{t}-\mu R_{l}. \end{array}$$

Here, *I_t_* represents the number of infected individuals on ART in the local population. The parameter *γ_t_* controls the rate at which infected individuals initiate ART and *ν_t_* the rate at which individuals on ART are removed from the infectious population, which is allowed to differ from the base removal rate *ν*.

The structured coalescent model corresponding to this epidemiological model has the transmission rate matrix:

(11) $$F(t)\;=\;\left(\begin{array}{lll} \beta_{e\to e}\frac{S_{e}}{N_{e}}I_{e} & \beta_{e\to l}(t)\frac{S_{l}}{N_{l}}I_{e} & 0\\ \beta_{l\to e}I_{l} & \beta_{l\to l}(t)\frac{S_{l}}{N_{l}}I_{l} & 0\\ \beta_{t\to e}I_{t} & \beta_{t\to l}(t)\frac{S_{l}}{N_{l}}I_{t} & 0 \end{array}\right).$$

The elements in *F* describe how lineages move between infected individuals in different populations through transmission events but does not take into account the movement of a lineage into the ART-infected compartment when a host initiates treatment. We therefore need to modify (6) above for tracking the lineage state probabilities to include the rates *g_kl_* at which lineages transition between states *k* and *l* independent of transmission events:

(12) $$\frac{dp_{ik}}{dt}=\sum_{l}\left(\frac{f_{kl}}{I_{l}}p_{il}+\frac{g_{kl}}{I_{l}}p_{il}-\frac{f_{lk}}{I_{k}}p_{ik}-\frac{g_{lk}}{I_{k}}p_{ik}\right)-p_{ik}\sum_{j\neq i}^{j\in A(t)}\sum_{l}^{m}\frac{f_{kl}+f_{lk}}{I_{k}I_{l}}p_{jl}.$$

Including these types of transitions is discussed in more detail by Volz (2012).

For our model with ART, all *g_kl_* = 0 except *g_lt_* which gives the rate at which lineages transition into the treatment class:

(13) $$G(t)\;=\;\left(\begin{array}{lll} 0 & 0 & 0\\ 0 & 0 & \gamma_{t}I_{l}\\ 0 & 0 & 0 \end{array}\right).$$

Parameters and prior distributions for the ART model are given in Table 5. In this model, we still estimate the time-varying transmission rates $\beta_{e\to l}(t)$ and $\beta_{l\to l}(t)$, but transmission from individuals on ART is parameterized in terms of a time-invariant scalar *κ_t_* of the time-varying local transmission rate: $\beta_{t\to l}(t)=\kappa_{t}\beta_{l\to l}(t)$.

**Table 5.** Parameters and prior distributions for model with ART.

Parameter	Name	Prior distribution	Prior values
κt	ART transmission scalar	Beta	α = 1.0; β = 1.0
γt	ART initiation rate	Log-normal	μ = −1.0996; σ = 0.5
νt	ART removal rate	Log-normal	μ = −2.2769; σ = 0.5

#### 2.7.4 AIDS-related deaths model

Our fourth model variant allows HIV-positive individuals to progress to an AIDS stage of infection with a higher death rate. Therefore, unlike in our other models where infected individuals are ‘removed’ from the infectious population but do not suffer increased mortality, AIDS-related deaths can permanently remove infected individuals from the entire population. This may be a more realistic way of modeling the demographic impact of the HIV epidemic, as there is strong evidence that AIDS-related deaths dramatically reduced adult lifespan before ART become widely available (Herbst et al. 2011; Bor et al. 2013). However, demographic surveillance in the AHRI study area indicates that the adult population size did not substantially change over this same time period. We therefore let net immigration into the local population demographically compensate for increased adult mortality due to AIDS. In our model, AIDS-related deaths are therefore directly offset by immigration from the external population.

The epidemic dynamics under this model are described by the following system of differential equations:

(14) $$\begin{array}{l} \frac{dS_{l}}{dt}=\mu N_{l}+\mu_{a}(t)I_{a}\frac{S_{e}}{N_{e}}-(\beta_{e\to l}(t)I_{e}+\beta_{l\to l}(t)I_{l}+\beta_{a\to l}(t)I_{a})\frac{S_{l}}{N_{l}}-\mu S_{l}\\ \frac{dI_{l}}{dt}=\mu_{a}(t)I_{a}\frac{I_{e}}{N_{e}}+(\beta_{e\to l}(t)I_{e}+\beta_{l\to l}(t)I_{l}+\beta_{a\to l}(t)I_{a})\frac{S_{l}}{N_{l}}-(\mu +\gamma_{a})I_{l}\\ \frac{dI_{a}}{dt}=\gamma_{a}I_{l}-\mu_{a}(t)I_{a}. \end{array}$$

Here, *I_a_* represents the number of infected individuals in the AIDS stage. The parameter *γ_a_* controls the rate at which infected individuals progress to AIDS. The AIDS-related death rate $\mu_{a}(t)$ is allowed to differ from the death rate *μ* in the general population and vary over time. AIDS-related deaths are instantaneously offset by immigration from the external population. What fraction of immigrants are susceptible or infected is determined by the susceptible and infected fractions in the external population.

The structured coalescent model corresponding to this epidemiological model has the transmission rate matrix:

(15) $$F(t)\;=\;\left(\begin{array}{lll} \beta_{e\to e}\frac{S_{e}}{N_{e}}I_{e} & \beta_{e\to l}(t)\frac{S_{l}}{N_{l}}I_{e} & 0\\ \beta_{l\to e}I_{l} & \beta_{l\to l}(t)\frac{S_{l}}{N_{l}}I_{l} & 0\\ \beta_{a\to e}I_{t} & \beta_{a\to l}(t)\frac{S_{l}}{N_{l}}I_{a} & 0 \end{array}\right).$$

To account for lineage movement between infected compartments that occurs independently of transmission, we can again use (12) to track the lineage state probabilities, with the state transitions rates:

(16) $$G(t)\;=\;\left(\begin{array}{lll} 0 & \mu_{a}(t)I_{a}\frac{I_{e}}{N_{e}} & 0\\ 0 & 0 & \gamma_{a}I_{l}\\ 0 & 0 & 0 \end{array}\right).$$

The transition rate $\mu_{a}(t)I_{a}\frac{I_{e}}{N_{e}}$ accounts for the movement of lineages into the local population through immigration of already infected individuals. The rate $\gamma_{a}I_{l}$ accounts for lineage movement due the progression of infected individuals to the AIDS stage.

Parameters and prior distributions for the AIDS model are given in Table 6. As in the ART model, the transmission rate from individuals in the AIDS stage is parameterized in terms of a time-invariant scalar *κ_a_* of the time-varying local transmission rate: $\beta_{a\to l}(t)=\kappa_{a}\beta_{l\to l}(t)$. Estimating the AIDS progression rate *γ_a_* as free parameter, we obtained extremely high rates of progression to AIDS resulting in unrealistic estimates of several other parameters. We therefore fixed $\gamma_{a}=0.25$, giving an average time from infection to AIDS of four years.

**Table 6.** Parameters and prior distributions for model with AIDS-related deaths.

Parameter	Name	Prior distribution	Prior values
Κt	ART transmission scalar	Beta	α = 1.0; β = 1.0
γt	ART initiation rate	Log-normal	μ = −1.0996; σ = 0.5
νt	ART removal rate	Log-normal	μ = −2.2769; σ = 0.5

## 4. Results

**Figure 2.**
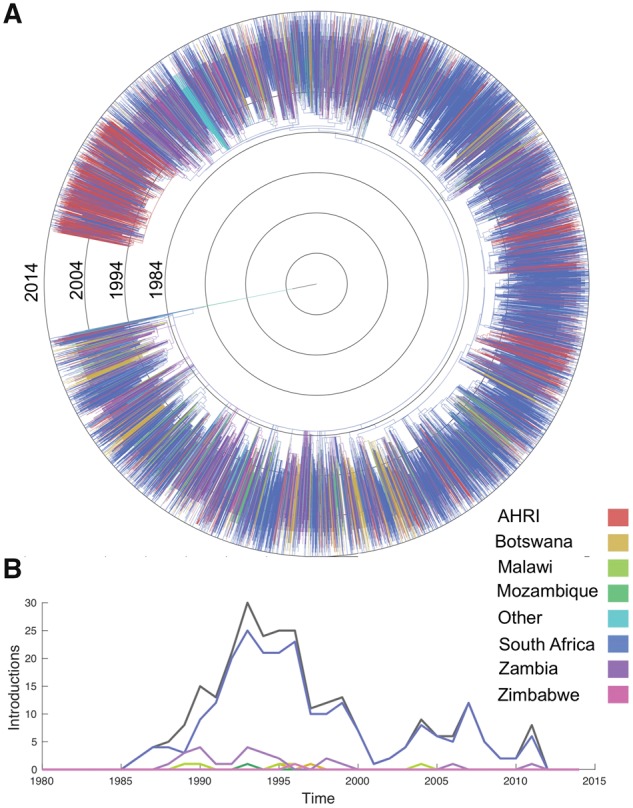
The local HIV epidemic in the AHRI study population within the larger phylogenetic context of the southern African subtype C epidemic. (A) ML phylogenetic tree reconstructed from HIV *pol* sequences from the AHRI (local) along with the regional background dataset. Tips are colored by sampling location, internal branches are colored according to their ancestral location reconstructed via maximum parsimony. **(**B) Time series showing the temporal distribution of external introductions from each country into the local population, as identified by maximum parsimony. The black line gives the total number of introductions summed over all countries.

To help situate the local epidemic in the AHRI study population within the larger context of the southern African HIV epidemic, an ML phylogeny was reconstructed from HIV-1 subtype C sequences sampled from 1,068 infected individuals in the AHRI population along with 11,289 sequences from a larger regional background dataset (Wilkinson et al. 2016) sampled throughout southern Africa (Fig. 2A). Branch lengths in the ML phylogeny were then rescaled into units of calendar time using least squares dating. Although there are some larger clades composed predominantly of local viral samples which likely represent locally evolving sub-epidemics, the majority of samples from the AHRI are interspersed throughout clades composed predominantly of external samples (i.e. from the regional background dataset), suggesting that many independent introduction events have occurred into the local population from elsewhere in South Africa or from other neighboring countries.

The ancestral location of each lineage in the ML tree was then reconstructed using maximum parsimony to reveal how viral lineages have moved over time (Fig. 2A). This allowed us to identify potential external introductions at branches in the phylogeny where the most parsimonious ancestral location transitioned from the external to the local population. Note that we define an external introduction as transmission from an individual in the external population to an individual living in the local population independently of whether the transmission event occurred inside or outside the local population because the phylogeny contains no information about the exact location of transmission. In total, 248 external introductions were identified into the local population. When transitions in ancestral location occurred between parent and child nodes, we used the midpoint between the parent and child node as a proxy for the timing of external introductions. Most of these presumed introduction events occurred between 1990 and 2000 during the period of rapid epidemic growth in South Africa, with relatively fewer introductions after 2000 once the epidemic stabilized (Fig. 2B). Most introductions occurred from elsewhere in South Africa, although a few potential introductions occurred from Botswana, Malawi, Mozambique, Zambia, and Zimbabwe.

**Figure 3.**
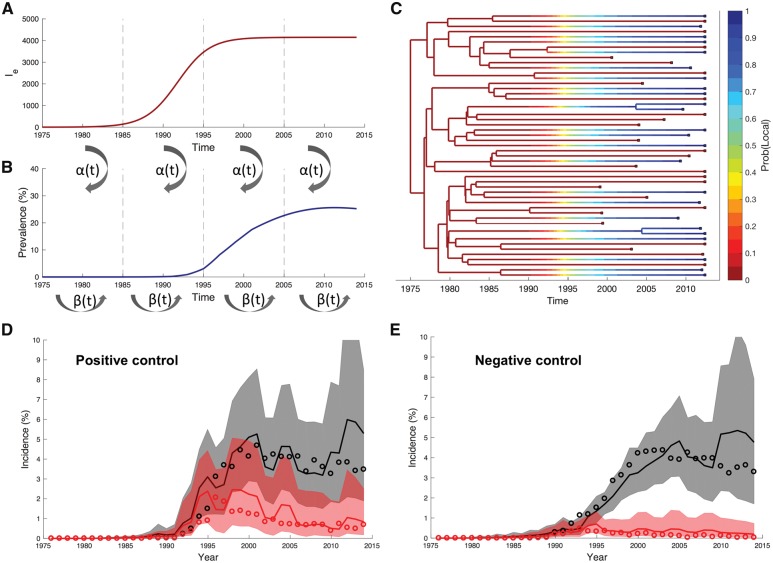
Schematic of the phylodynamic model and its validation on simulated data. Epidemic dynamics simulated under the model showing the number of infected individuals in the external population *I_e_* (A) and the local population (B). Transmission events from the external to the local population occur at rate α(t) and within the local population at a rate proportional to β(t). Both of these rates are time dependent and vary in a piecewise constant manner to accommodate changes in behavior, treatment or other interventions. Although not shown here, viral lineages can also be exported from the local population through transmission to the external population. (C) A simulated phylogeny generated under the same phylodynamic model. Each lineage is colored according to its probability of being in the local population (blue). These probabilities were computed under the model based on each lineage’s sampling location and the estimated transmission rates between populations. (D and E) Total incidence (gray) and incidence attributable to external introductions (red) inferred from simulated phylogenies. Solid lines represent the posterior median estimate, shaded regions mark the 95 per cent credible intervals and open circles mark the true yearly incidence known from the simulations. In the positive control (D), we correctly infer that external introductions played a large role in driving and sustaining the local epidemic; whereas in the negative control (E), we correctly infer that external introductions only played a minor role in seeding the epidemic.

Because phylogenies only contain lineages ancestral to sampled viruses, the number of external introductions identified in the foregoing analysis likely represents only a small fraction of the total number. Moreover, due to the large size of the epidemic relative to the number of infected individuals sampled (∼8%), it is extremely unlikely that we would have sampled both descendants of any recent transmission event between an external donor and a local recipient. In fact, the most recent common ancestor of most pairs of sampled viruses predates the early stages of the South African epidemic (Fig. 2A). The timing of reconstructed state changes may therefore not be a reliable proxy for the timing of introduction events since introductions may have occurred more recently (see Fig. 3C). It is highly likely then that our preliminary analysis based on parsimony both underestimates the true number of external introductions and skews their temporal distribution towards the more distance past.

### 4.1 Phylodynamic analysis

We therefore developed a phylodynamic model based on a simple but still realistic epidemiological model using a previously described structured coalescent framework (Volz 2012; Müller et al. 2017) in order to quantify the contribution of external introductions versus local transmission. The model tracks the number of infected individuals in the external population along with local epidemic dynamics (Fig. 3A and B). The model also probabilistically tracks how lineages in the tree move between populations based on their sampling location and the estimated transmission rates between populations (Fig. 3C). Tracking the movement of lineages in this way allows us to estimate whether new infections were derived from a local or external source. We validated our model using phylogenies simulated to reflect different epidemic scenarios. In the first scenario, external introductions play a large role in driving and sustaining the local epidemic (positive control). In the second scenario, external introductions only play a minor role in seeding the epidemic (negative control). In both scenarios, we were able to accurately estimate both the overall epidemic dynamics and the incidence attributable to internal and external transmission (Fig. 3D and E).

**Figure 4.**
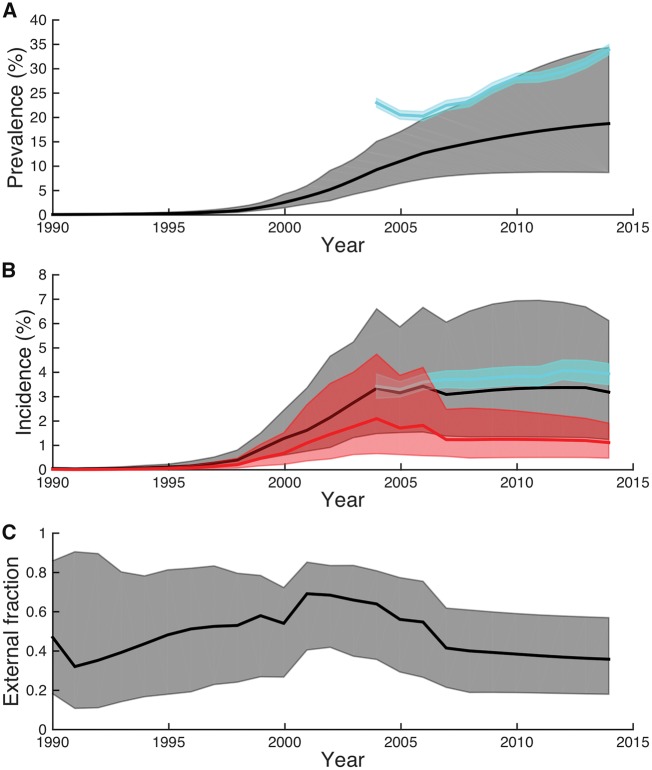
Epidemic dynamics reconstructed from viral phylogenies using the phylodynamic model. (A) Prevalence estimates from the phylogeny (gray) and independent surveillance data (blue). (B) Total incidence estimated from the phylogeny (gray) and surveillance data (blue). Incidence attributable to external introductions estimated from the phylogeny is shown in red. (C) The fraction of incidence attributable to external introductions over time. All solid lines represent the posterior median estimates while shaded regions mark the 95 per cent credible intervals. All estimates represent a posterior average over a set of phylogenies reconstructed from different sub-sampled datasets and thus take into account both phylogenetic uncertainty and sampling variance.

Using the phylodynamic model, we reconstructed epidemic dynamics in the local population from phylogenies reconstructed from the viral samples. As expected, both prevalence and incidence rapidly grew during the 1990s in the local population, and then grew more slowly after 2004 (Fig. 4A and B). Posterior estimates of the external infected population size through time and all inferred parameters are shown in Supplementary Figs. S1–S4. After 2004, independent estimates of prevalence and incidence based on population surveillance data are available from the AHRI. Prevalence estimates based on surveillance data were about 10 per cent higher than our phylodynamic estimates, although both methods estimate a similar growth in prevalence since 2004 when widespread access to ART began (Fig. 4A), consistent with earlier reports by (Zaidi et al. 2013). Estimates of incidence since 2004 are in closer agreement, with both methods returning a median estimate of yearly incidence between 3 and 4 per cent with no detectable decline since ART coverage began increasing in 2004 (Fig. 4B).

Given that we could reliably reconstruct overall epidemic dynamics from simulated and empirical viral phylogenies, we used the phylodynamic model to quantify the relative contribution of external introductions versus local transmission to overall incidence. During the earliest stages of the local epidemic, the incidence attributable to external introductions was very high (Fig. 4B, red). While after 2004 the fraction attributable to external introductions declined, as of 2014 an estimated 35 per cent [95% confidence interval (CI): 20–60%] of all present day infections were due to external introductions (Fig. 4C).

### 4.2 Model testing and robustness

While our phylodynamic reconstruction of epidemic dynamics appears consistent with population-based surveillance, our model makes several simplifying assumptions about HIV’s transmission dynamics and demography in the AHRI population. We therefore formulated four different variants of the base model used above to relax what we view as the most questionable of these assumptions. We then fit each variant to a single ML phylogeny to gauge how robust our phylodynamic estimates were to relaxing these assumptions. Informal comparisons of the marginal likelihood of the phylogeny under each model showed that all but one variant fit the phylogeny better than the base model (Table 7). There were also interesting differences in the epidemic dynamics reconstructed under each model as we discuss below.

**Table 7.** Comparison of phylodynamic model fit and epidemiological estimates as of 2014.

Model	Marginal log likelihood	Prevalence (%)	Incidence (%)	Fraction external
Base	−18,278	21	2.37	0.35
Hot spots	−18,229	21.3	2.6	0.31
txRemoval	−18,282	27.6	3.61	0.21
ART	−18,255	35.1	4.82	0.2
AIDS	−18,239	47	5.1	0.05 (+0.31 migrants)

Values reported are median posterior estimates.

**Figure 5.**
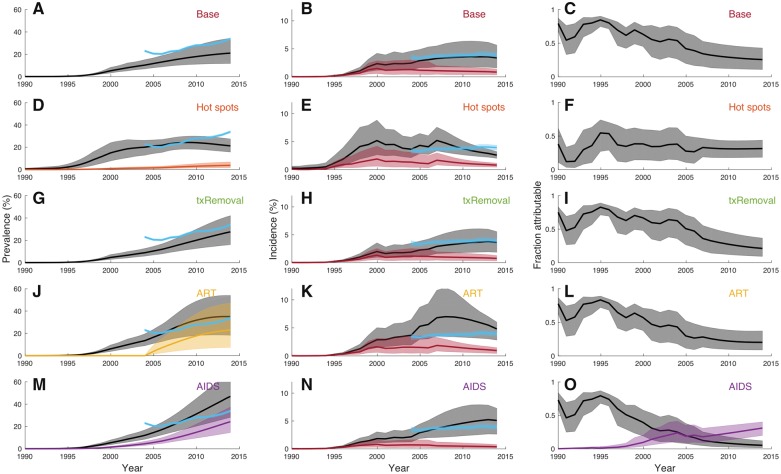
Epidemic dynamics reconstructed under different variants of the phylodynamic model. (A–C) Estimates from fitting the base model to the same ML phylogeny as the other model variants. (D–F) Model with geographic hot and cold spots of prevalence. Estimated prevalence in hot spots as a percentage of the total population size is shown in orange. (G–I) Model with time-varying removal rates. (J–L) Model that included antiretroviral treatment after 2004. Prevalence of infected individuals on ART is shown in gold. (M–O) Model with AIDS-related deaths and migration from external population. Prevalence of AIDS is shown in purple. (O) The fraction of new cases attributable to immigration of already infected individuals is also shown in purple. As before, the fraction of new cases attributable to transmission from the external population is shown in gray. Prevalence and incidence estimates from population-based surveillance (blue) are replicated here for easy comparison with Fig. 4.

The first model variant included geographic hot and cold spots of prevalence, relaxing the assumption in the base model of no population substructure within the AHRI population. Considering this form of subpopulation structure is a natural choice because the AHRI population is clustered into areas of high prevalence (>25%) along major roads and areas of low prevalence (<10%) in more inaccessible rural areas (Tanser et al. 2009) (Fig. 1C). While adding subpopulation structure substantially increased the marginal likelihood relative to the base model (Table 7), prevalence is still underestimated relative to population-based surveillance and incidence is also estimated to be slightly lower (Fig. 5D and E). Nevertheless, our estimates of the fraction of incidence attributable to external introductions are very similar to those estimated under the base model (Fig. 5F).

Because prevalence was underestimated in the base and hot spot model, we considered a second model where individuals could remain in the infected class longer due to removal rates decreasing over time. Adding time-varying removal rates to the base model did result in a more realistic increase in prevalence towards present, but total prevalence was still underestimated relative to population-based surveillance (Fig. 5G). Adding time-varying removal rates did not increase the marginal likelihood relative to the base model, but unlike the other model variants this model did not add an additional population state to the phylodynamic model.

Because allowing for a longer duration of infection appeared to improve our estimates of prevalence, we considered a third model where infected individuals could initiate ART after 2004. Under this model, ART was allowed to not only increase the duration of infection but also reduce transmissibility for individuals on ART. Interestingly, prevalence estimates become more consistent with population-based surveillance under this model while total incidence was overestimated towards present (Fig. 5J and K). Estimates of the fraction attributable to external introductions were only slightly lower than under the base model (Fig. 5L, Table 7).

Finally, the fourth model included progression to a late stage of infection or AIDS with an increased death rate, consistent with observations that life expectancy dropped significantly during the early stages of the KZN epidemic before ART become widely available (Bor et al. 2013). However, an increased AIDS-related death rate would cause the adult population size to decline over time, inconsistent with demographic surveillance data from the AHRI study area. We therefore allowed AIDS-related deaths to be offset by immigration from the external population to keep the adult population size constant. Because there is no evidence that in-migration was significantly higher than out-migration this is a rather extreme assumption, but it allowed us to test our results against the assumption that there was no direct migration of already infected individuals into the local population. Under this model, phylodynamic estimates of prevalence were substantially higher than under other models, largely due to already infected individuals migrating into the local population (Fig. 5M). We also estimated a relatively lower fraction of incidence due to transmission from the external population (Fig. 5N, Table 7). However, this is not necessarily inconsistent with our other estimates. Fewer external introductions due to transmission from the external population were simply offset by immigration of already infected individuals into the local population (Fig. 5O), which can be viewed as another source of external introductions.

## 5. Discussion

Our phylodynamic results on external introductions fit within a growing body of evidence that transmission networks are highly interconnected across communities even at relatively large geographic distances due to human movement patterns. Historians and social scientists have long noted that both mass migration and increased mobility may have played an important role in the rapid spread of HIV through southern Africa (Iliffe 2006). In South Africa, in particular, historical patterns of circular labor migration among neighboring countries and increased mobility following the end of the Apartheid system may have contributed to the rapid growth of the epidemic (Jochelson et al. 1991; Lurie et al. 1997; Hargrove 2008). More recently, phylogenetic studies of HIV in Africa have provided support for frequent viral movement at spatial scales ranging from local communities (Grabowski et al. 2014) to across national borders (Wilkinson et al. 2016). This frequent movement has been linked to highly mobile individuals such as economic migrants, soldiers, sex workers, and truck drivers (Gray et al. 2009; Wilkinson et al. 2016).

Our phylogenetic and phylodynamic analysis of the HIV epidemic in the Africa Health Research Institute study population revealed that external introductions via human movement played and continue to play a vital role in driving the epidemic in rural KZN. A preliminary phylogenetic analysis based on maximum parsimony suggested that a large wave of introductions occurred in the 1990s during the early stages of the South African epidemic. A subsequent analysis using a more realistic phylodynamic model confirmed that the earliest stages of the epidemic were indeed largely driven by external introductions, although these early introductions may have been less wave-like.

Our phylodynamic analysis also suggested that, far from just seeding the local epidemic, external introductions continue to play an important role in sustaining the high incidence of HIV in local KZN populations. This has direct relevance for ART as prevention (TasP) trials and programs. If most transmission occurs locally, TasP programs should be able to efficiently and cost-effectively reduce incidence (Granich et al. 2009) and could selectively target communities with higher incidence (Tanser et al. 2009). However, if most new infections are acquired externally, then increasing local ART coverage may not substantially reduce incidence. Our phylodynamic estimates for one KZN population indicate that the situation likely lies between these two extremes. Our median estimate is that presently 35 per cent (95% CI: 20–60%) of new infections in the AHRI population are attributable to external introductions, suggesting a substantial number of new infections could be prevented if the source of these infections could also be targeted. Nevertheless, these results imply that the majority of new infections may be attributable to local transmission, and increasing local ART coverage may still prevent many future infections. However, a recent TasP trial in a population immediately adjacent to the AHRI study area showed no incidence reduction despite universal access to ART (Iwuji et al. 2017). Identifying the source of new infections and whether they arose locally from untreated infections or from external introductions will be key to understanding why this trial failed to decrease incidence.

While phylogenies can reveal the movement of viruses between populations, they cannot necessarily reveal where transmission events occurred or how viral lineages first entered a population without additional information about human movement. An external introduction event may result from a visitor transmitting to an individual residing in the local population or while an individual currently residing in the local population was living away or traveling. In our phylodynamic model, we cannot distinguish between these two scenarios because they result in identical phylogenetic patterns. The AHRI does however collect data on the residence and migration status of individuals in the study area. Younger men and women frequently leave and return to the area, and those who leave spend a substantial amount of their time (30.8%) outside the area (Dobra et al. 2017). About 55 per cent of these migration events occur within a 300-km radius of the study area and generally fall within the province of KZN, including Richards Bay and Durban (Dobra et al. 2017). Furthermore, these data show that prevalence is higher among residents with histories of recent migration (McGrath et al. 2015). Individuals who spend more time away and travel longer distances outside their community also have a significantly higher risk of acquiring HIV infection (Dobra et al. 2017). Both of these observations support the idea that local residents may be infected while living or traveling outside of the study area and then return to the area, highlighting the potential importance of circulatory migration patterns. The extent to which external introductions are occurring outside local communities could be further teased apart by combining viral phylogenetics with detailed sociological data on the migration history of sampled individuals to infer the location and migration status of individuals at the time of infection.

Although phylodynamics is increasingly used to study epidemic dynamics, the validity of estimates derived entirely from viral phylogenies can of course be questioned and have their own limitations as just discussed. We however feel confident that our main results about external introductions are reliable for several reasons. First, we validated our model on simulated phylogenies and found that we could accurately reconstruct total incidence and the fraction of incidence attributable to external introductions in scenarios where introductions both did and did not play a large role in driving epidemic dynamics. Second, we were able to reconstruct dynamics consistent with prevalence and incidence trends estimated from independent surveillance data. Third, while there is no ‘gold standard’ to which we can compare our phylodynamic estimates of external introductions, our estimated fraction of external introductions is consistent with known mobility patterns in the AHRI population where 38 per cent of males and 32 per cent of females are recent migrants or frequently leave the area (Camlin et al. 2010; Muhwava et al. 2013). Finally, our estimates of external introductions appear robust to the exact formulation of the epidemiological model assumed. Different variants of our model including prevalence hot spots, time-varying removal rates, ART or AIDS-related deaths all returned similar estimates of external introductions.

In addition to showing that external introductions play an important role in sustaining high HIV incidence in this hyper-epidemic setting, we were also able to accurately estimate population-level incidence. We believe that this is also an important result of our study, as there is currently great interest in using phylodynamics to quantify epidemic dynamics, especially changes in incidence, without the need for expensive longitudinal cohort studies (Dennis et al. 2014; Cohen 2015). A recent simulation study has demonstrated that it may be possible to estimate incidence dynamics from phylogenies in the context of African HIV epidemics (Ratmann et al. 2017), but the accuracy of estimates varied considerably depending on the choice of phylodynamic model, with the most complex structured coalescent model vastly outperforming other models. In contrast, we accurately estimated incidence and, to a lesser extent, prevalence from empirical phylogenies using relatively simple models.

In this respect, we view the simplicity of our model as one of its strengths, even though far more detailed epidemiological models have been developed in recent years for the purposes of predicting and evaluating the impact of HIV prevention efforts (see Eaton et al. 2012 for a review). Our desire for simplicity was motivated by the fact that pathogen phylogenies are only a proxy for the unobserved transmission tree, and only contain information about a subset of transmission events from which we can draw inferences about epidemic dynamics. By itself then, a pathogen phylogeny contains no information about the underlying drivers of transmission. We therefore opted to make minimal mechanistic assumptions about how changes in risk behavior, prevention efforts, and clinical care all interact to shape HIV’s complex transmission dynamics. Adding parameters or state variables to our model in order to capture these processes may reduce certain biases, but will inevitably increase the variance of our phylodynamic estimates since the phylogeny contains no information about them.

Nevertheless, it remains largely unexplored what epidemiological factors need to be included in phylodynamic models in order for estimates to be accurate and robust to slight model misspecifications. This is especially true when we try to relate population-level variables of interest like prevalence or incidence back to the branching structure of a phylogeny since many processes simultaneously shape the phylogeny and may confound analysis if not properly accounted for. Previous work has shown that major subdivisions within a host population can distort inferences because lineages in different subpopulations are no longer exchangeable as standard coalescent theory assumes (Heller et al. 2013; Rasmussen et al. 2014a). We therefore accounted for subdivision between local and external populations using a structured coalescent model (Volz 2012). But it is less clear what other forms of host heterogeneity need to be considered for HIV. For example, contact heterogeneity in sexual networks can also shape pathogen phylogenies, increasing the coalescent rate during the early stages of an epidemic as the pathogen spreads though highly connected parts of the network (O'Dea and Wilke 2011; Leventhal et al. 2012; Rasmussen et al. 2017). It is therefore possible that if we had accounted for contact heterogeneity, we may have estimated a higher incidence during the early stages of the epidemic in the AHRI population, bringing our estimates of prevalence closer to those observed when population-based surveillance began in 2004.

For a chronic infection like HIV, it may also be important to correctly model the progression of individuals though different stages of infection or clinical care. Phylodynamic models for HIV that allow for disease progression have been shown to accurately reconstruct epidemic dynamics from real and simulated data (Volz et al. 2013; Ratmann et al. 2017). In our case, adding an infection class for people on ART corrected our underestimates of prevalence by allowing individuals on ART to remain infected longer. But while it is conceptually straightforward to model multiple stages of infection, transmission and progression rates for each of these stages can also vary over time, leading to very parameter-rich models. As a first-order approximation, we therefore tried to circumvent this complexity by only modeling temporal variability in transmission rates independent of disease progression. However, this strategy proved insufficient to capture realistic changes in prevalence and it was necessary to include either time-varying removal rates or an ART-infected class to account for increasing durations of infection after the rollout of ART in 2004. Encouragingly though, our estimates of incidence were surprisingly accurate even under the base model, suggesting that highly detailed models may not be necessary to estimate incidence accurately. We suggest that this may be due to the fact that, while simple, our base model already includes two of the most relevant features of HIV’s transmission dynamics in rural Africa: time-varying transmission rates and local versus external contact structure.

In conclusion, we believe our results demonstrate the power of using phylodynamics to study HIV transmission dynamics in large, generalized African epidemics. Using a relatively simple phylodynamic model, we were able to quantify the relative contribution of external introductions to address a longstanding question about the role human mobility plays in local HIV epidemics, while showing for the first time that it is possible to accurately estimate HIV incidence from empirical data in the context of generalized African epidemics. Given the promising performance of our phylodynamic model, we have made it freely available as an add-on to the widely used phylogenetic software package BEAST 2 (Bouckaert et al. 2014; Rasmussen 2017). Finally, we note that as HIV sequence datasets expand to encompass larger spatial regions, it should be possible to use similar phylodynamic approaches to quantify transmission rates between multiple different communities and thereby pinpoint the geographic source of new infections—providing detailed knowledge that can be used directly to prevent new infections.

## Supplementary Material

Supplementary Figure 1

Supplementary Figure 2

Supplementary Figure 3

Supplementary Figure 4
