# Supplementary figures and images for: Tracking external introductions of HIV using phylodynamics reveals a major source of infections in rural KwaZulu-Natal, South Africa
Source: Virus Evol. 2018 Dec 11;4(2):vey037. doi: 10.1093/ve/vey037 (PMC6290119; doi:10.1093/ve/vey037)

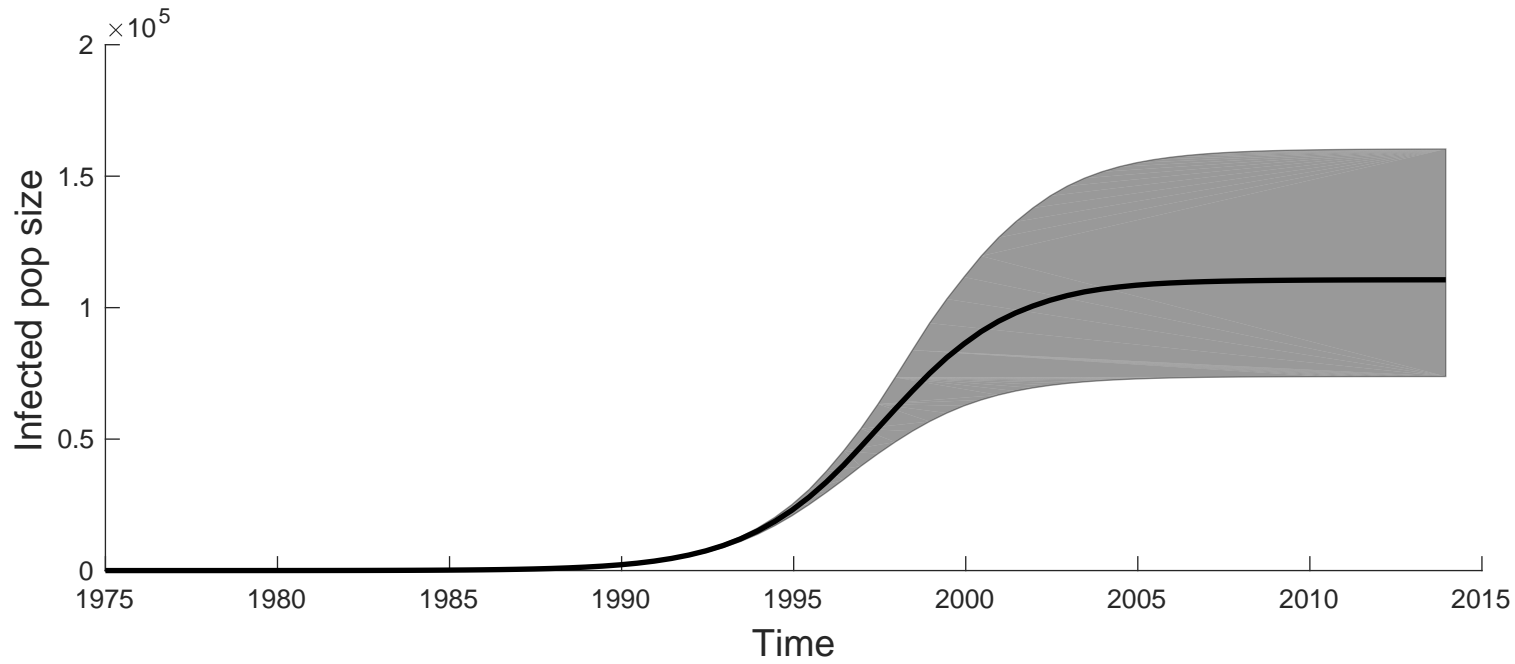

Supplement: Supplementary Figure 1 [file vey037_supp_figs1.pdf]

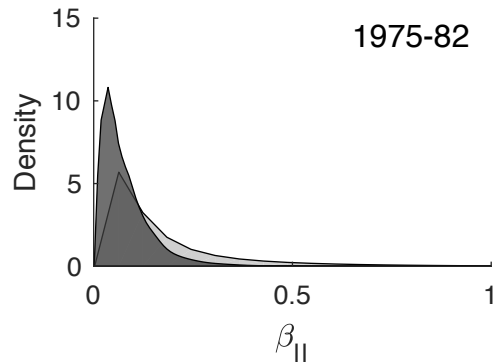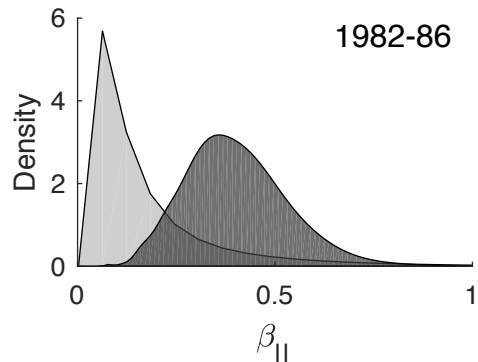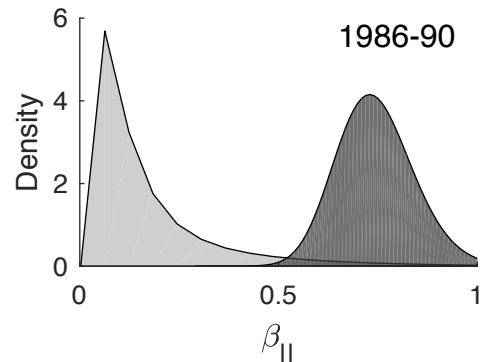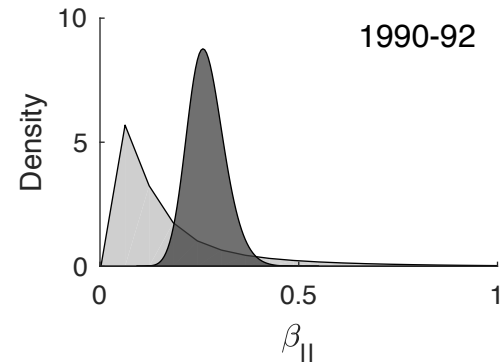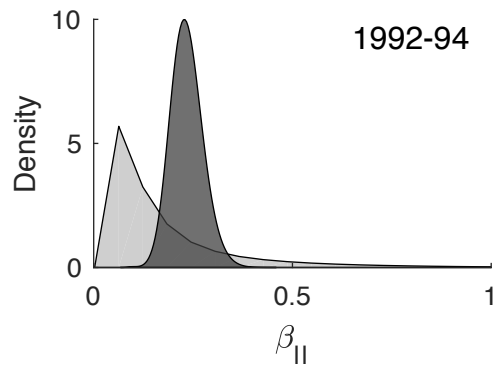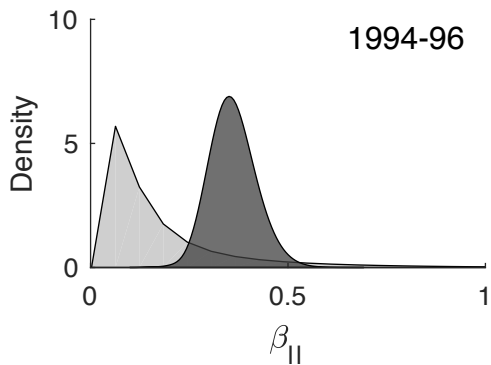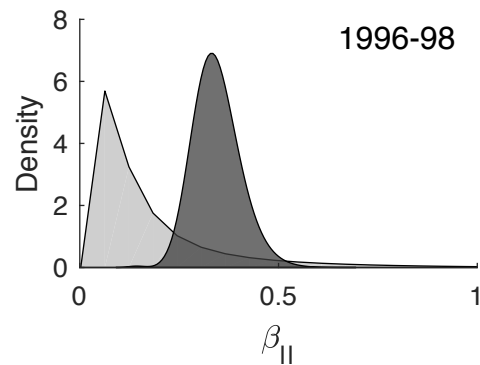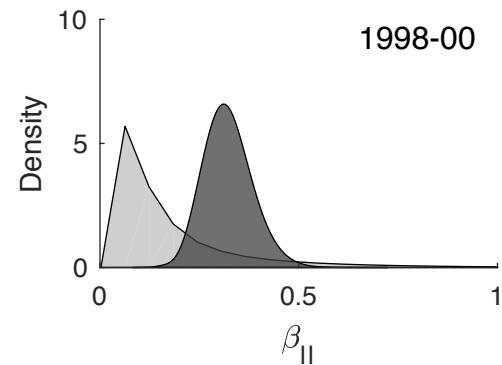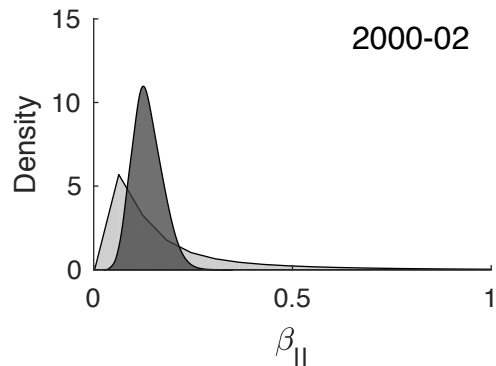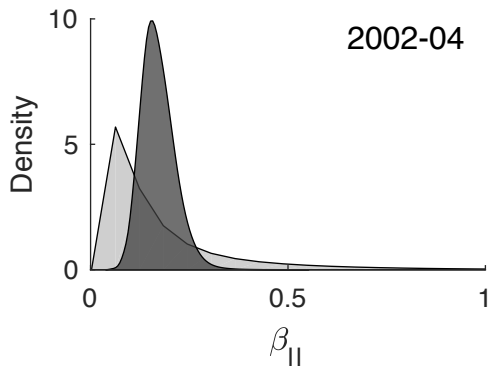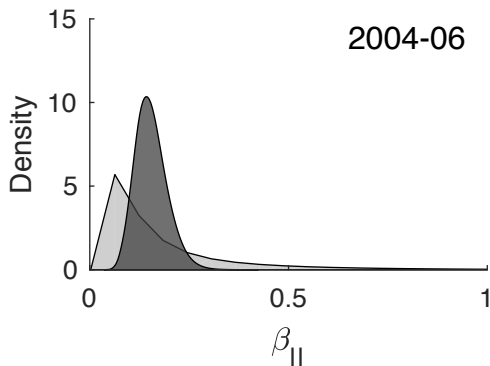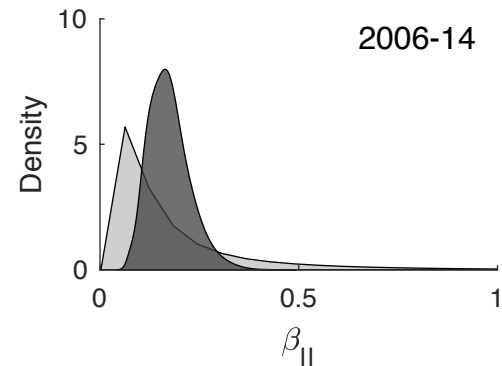

Supplement: Supplementary Figure 2 [file vey037_supp_figs2.pdf]

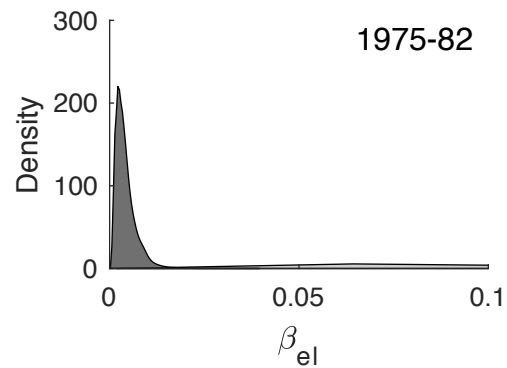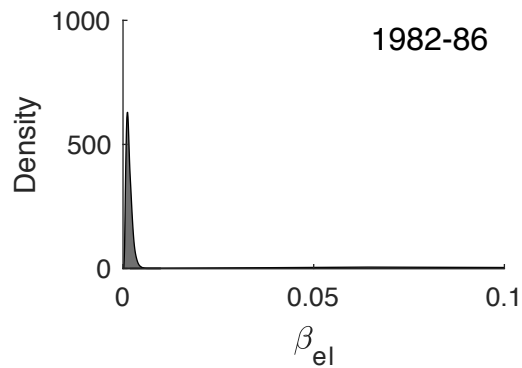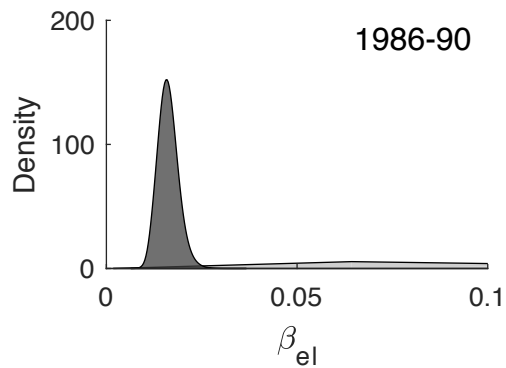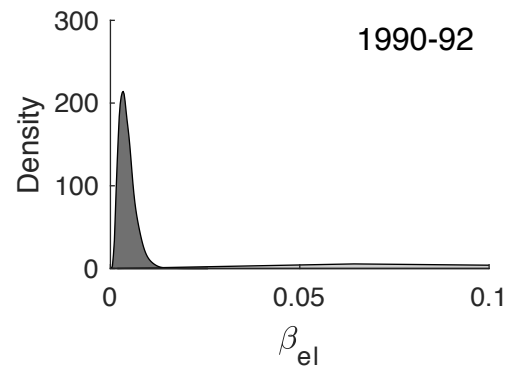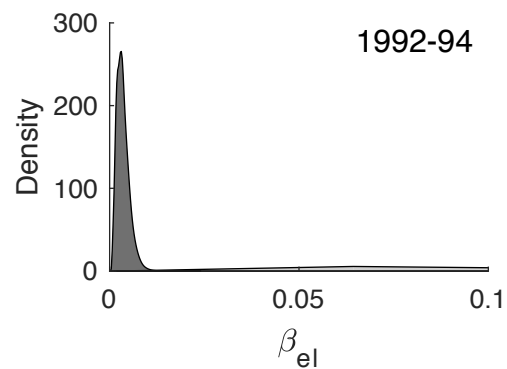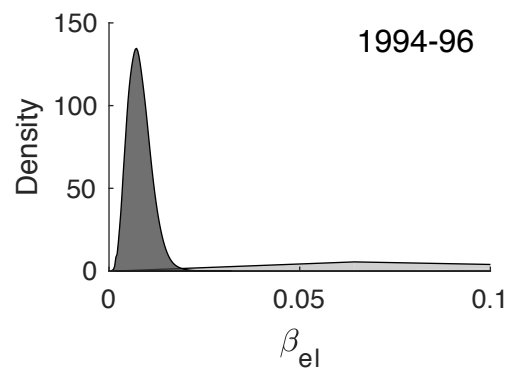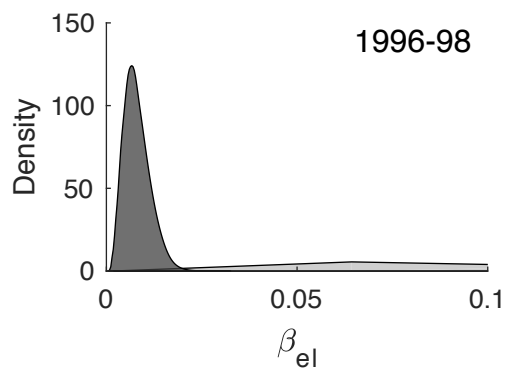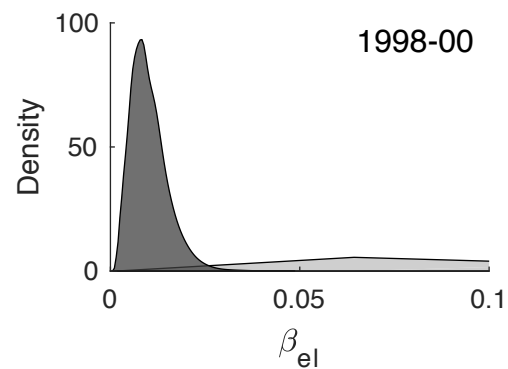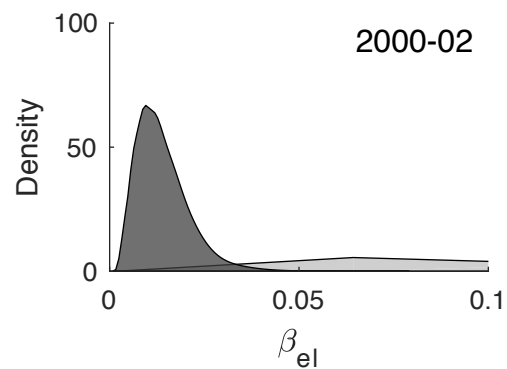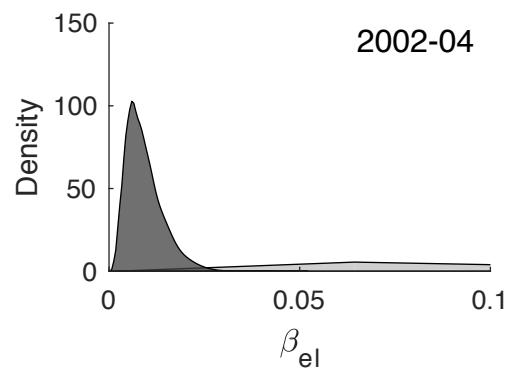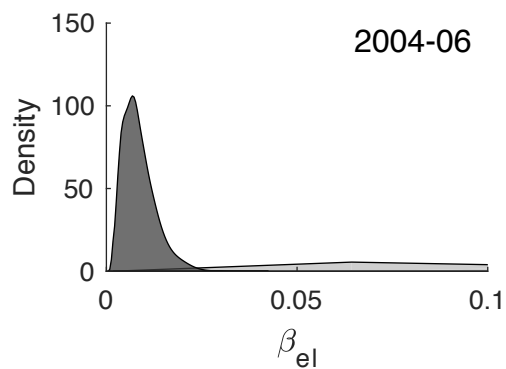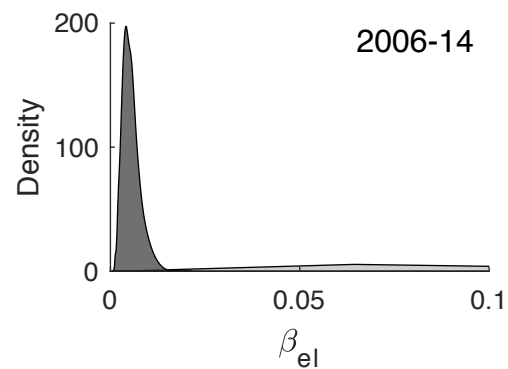

Supplement: Supplementary Figure 3 [file vey037_supp_figs3.pdf]

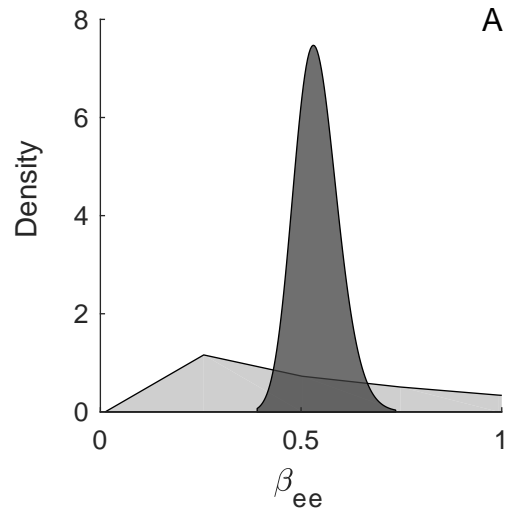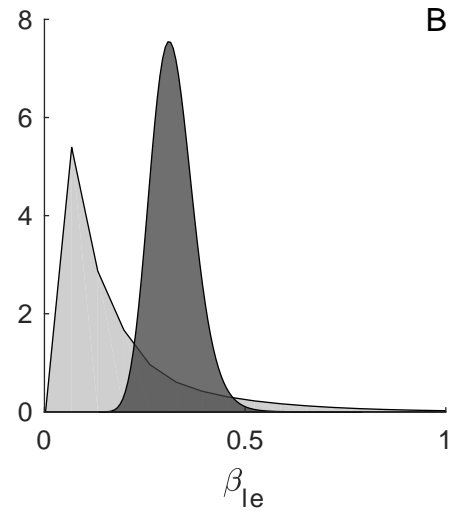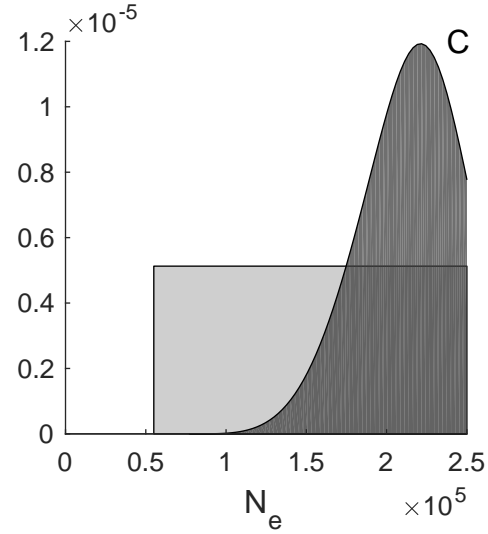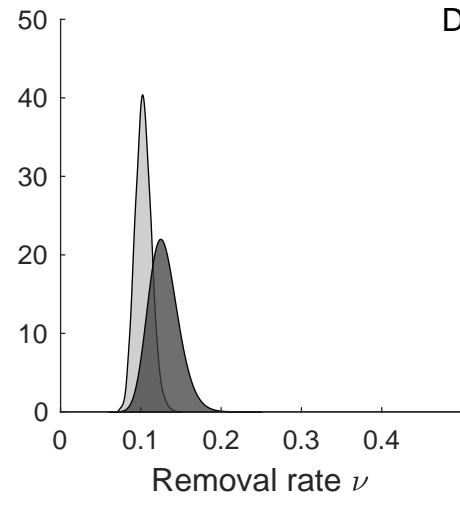

Supplement: Supplementary Figure 4 [file vey037_supp_figs4.pdf]
